# An Optical Signal Simulator for the Characterization of Photoplethysmographic Devices

**DOI:** 10.3390/s24031008

**Published:** 2024-02-04

**Authors:** Erika Pittella, Orlandino Testa, Luca Podestà, Emanuele Piuzzi

**Affiliations:** 1 Department of Information Engineering, Electronics and Telecommunications (DIET), Sapienza University of Rome, 00184 Rome, Italy; orlandino.testa@uniroma1.it (O.T.); emanuele.piuzzi@uniroma1.it (E.P.); 2 Department of Astronautical, Electrical and Energy Engineering (DIAEE), Sapienza University of Rome, 00184 Rome, Italy; luca.podesta@uniroma1.it

**Keywords:** characterization of biomedical devices, optical signal simulator, monitoring of vital signs

## Abstract

(1) Background: An optical simulator able to provide a repeatable signal with desired characteristics as an input to a photoplethysmographic (PPG) device is presented in order to compare the performance of different PPG devices and also to test the devices with PPG signals available in online databases. (2) Methods: The optical simulator consists of an electronic board containing a photodiode and LEDs at different wavelengths in order to simulate light reflected by the body; the PPG signal taken from the chosen database is reproduced by the electronic board, and the board is used to test a wearable PPG medical device in the form of earbuds. (3) Results: The PPG device response to different average and peak-to-peak signal amplitudes is shown in order to assess the device sensitivity, and the fidelity in tracking the actual heart rate is also investigated. (4) Conclusions: The developed optical simulator promises to be an affordable, flexible, and reliable solution to test PPG devices in the lab, allowing the testing of their actual performances thanks to the possibility of using PPG databases, thus gaining useful and significant information before on-the-field clinical trials.

## 1. Introduction

Heart disease poses a significant global public health challenge and stands as the primary cause of death worldwide, particularly prevalent in large urban populations. The World Health Organization reported 17.3 million deaths attributable to heart disease in 2012, a number projected to escalate to 23.6 million by 2030 [1]. The escalating mortality rates underscore the imperative focus on preventing cardiovascular diseases and early diagnosis within contemporary cardiology.

Traditionally, clinical studies address cardiovascular issues post-manifestation. To proactively mitigate the risk of cardiovascular diseases, one effective monitoring approach utilizes photoplethysmography (PPG) [2]. Wearable devices equipped with PPG technology detect changes in vessel blood volume, offering a non-invasive method to explore heart rate, cardiovascular diseases, respiratory rate, blood oxygen saturation, autonomic functions, and tissue perfusion [3,4,5,6,7,8,9,10]. The assessment of human physiological parameters through photoplethysmographic signals recorded by portable PPG devices is a current and significant scientific pursuit [11,12]. Portable PPG devices, serving as alternatives to electrocardiography (ECG) devices, are highly sought after in the medical field due to their versatility, non-invasiveness, and cost-effectiveness [13].

To date, many studies are being conducted on these promising technologies [14,15,16]; this work aims to contribute to this research topic. In particular, in a previous paper, an experimental setup was developed, making use of the prototype of an artery model obtained by modifying an emulator developed and available at the Microwaves and Electromagnetic Compatibility Laboratory of the Sapienza University of Rome [17,18]. This system allows us to compare, in a repeatable and controlled way, the acquisitions of different PPG devices, and it opens up the possibility of comparing and analyzing signals from other wearable technologies [19]. However, the artery model has some deficiencies, such as, for example, the difficulty in using signals coming from medical databases, a certain complexity in generating arbitrary signals, and, in general, some problems in employing PPG signals characterized by a high heart rate due to the inherent slow response of the hydraulic setup.

In this paper, an optical simulator suitable to provide a perfectly reproducible signal with the desired characteristics as an input to a PPG device is presented in order to make the study and characterization of PPG devices more robust and complete. In fact, in this way, it is possible to compare the performance of different PPG devices, modifying the amplitude of the input signal to characterize the device sensitivity and also to use PPG signals taken from online databases and, therefore, belonging to subjects with a range of different characteristics (physiological and pathological). The optical simulator consists of an electronic board containing a photodiode and some LEDs at different wavelengths to reproduce the part of light radiation reflected by the body that the PPG device expects to receive. In fact, the PPG device sends light radiation toward the subject’s skin, and the photoplethysmographic signal is obtained from the part of the radiation reflected by (or transmitted through) the tissue [20]. The PPG signal taken from the chosen database is sent to the designed electronic board using the NI USB 6361 DAQ card [21], which is controlled via software using a LabVIEW VI (LabVIEW 2013) [22]. The used wearable medical device is an earphone [23], which allows for the monitoring of internal body temperature, heart rate, and blood oxygen saturation. To characterize this device, a PPG signal from a healthy subject taken from a database was used, whose amplitude was appropriately varied to evaluate the sensitivity threshold of the device itself. The repetition rate of the signal was also artificially varied in order to test the response of the PPG device to variations in heart rate. Finally, in-depth studies were also conducted on the optical simulator and, in particular, on the LEDs, whose emitted power was evaluated as a function of the voltage average value at the input; for these measurements, an optical power meter was used [24,25].

## 2. Materials and Methods

### 2.1. PPG Signals

The photoplethysmographic technique is based on the proportionality between the quantity of transmitted or reflected light radiation and the changes in blood volume in the microvascular system; in fact, the waveform of the PPG signal describes how the attenuation of light radiation varies when it is transmitted or reflected by biological tissues [1]. This light that penetrates the skin and blood vessels is then captured by a photodetector to measure blood flow. The radiation that returns to the photodetector, and therefore the PPG signal itself, depends on the flow of blood and oxygen that passes through the capillaries with each heartbeat.

**Figure 1:**
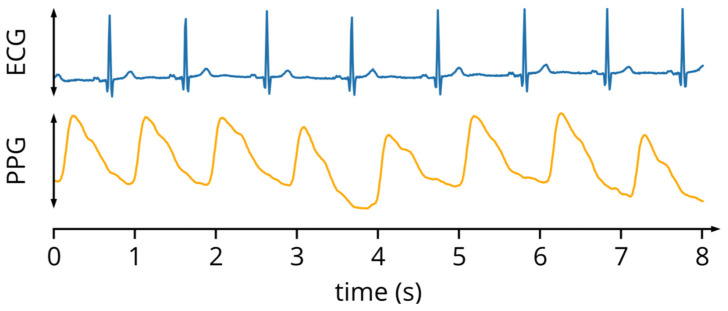
ECG and PPG signals.

In the PPG signal, two main components can be distinguished: the pulsatile one, often called the “AC” component, and a continuous component higher than the previous one, known as “DC”. The pulsatile component varies its amplitude with each cardiac cycle (Figure 1), as in the systolic phase, there is a volume of blood in the microvascular tissue that is different from that of the diastolic phase. In particular, when the blood volume increases, the light radiation is more attenuated, and this implies that the reflected radiation is inversely proportional to the filling state of the vessels, so it is minimum in conditions of maximum capillary filling and maximum in conditions of maximum capillary emptying. The pulsatile component is, therefore, of the greatest interest as it reflects the movement of the blood volume in the vessel, which varies with the heart rate (see Figure 1) and, therefore, has a fundamental frequency typically around 1 Hz.

### 2.2. Cosinuss° Two

**Figure 2:**
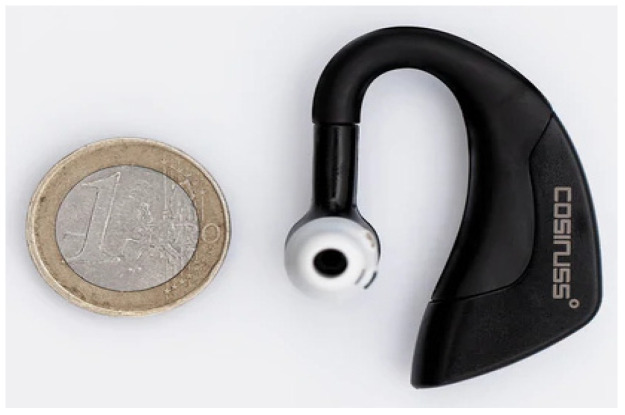
A picture of the cosinuss° Two.

The PPG device used as an example to validate the developed optical emulator is the cosinuss° Two, shown in Figure 2, a device born from the idea of introducing remote monitoring of patients in domestic isolation and affected by COVID-19 with medium–low progression of infection [23]. It is designed to detect, analyze, and transmit information regarding the physiological signals of the body, such as vital signs and environmental data, and provide immediate biofeedback to the wearer and/or remote healthcare staff, collecting a large variety of raw data [19,23]. The wearable sensor measures vital signs from inside the ear canal and transmits the data via Bluetooth to cosinuss° LabGateway. The gateway forwards the data to a telecommunications network, which sends it to cosinuss° LabServer. Through the web interface, healthcare professionals can view and access the vital information of each patient [23].

### 2.3. PPG Device Characterization

**Figure 3:**
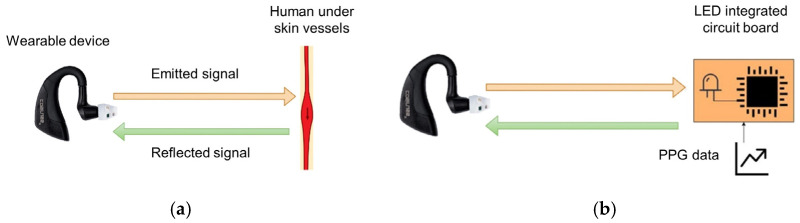
Scheme of the conventional use of a PPG device (**a**) and the one proposed in this paper (**b**).

The device characterization is conducted using an optical simulator that reproduces the PPG signal reflected by the body starting from values taken from a database instead of using the device in contact with the subject (Figure 3a). This approach, schematized in Figure 3b, has the following advantages:

- Use of recorded physiological PPG signals, which allow effective testing of diagnostic capabilities of the device;
- Repeatability of the generated signal, allowing comparisons between different PPG sensors;
- The PPG signal software generation allows us to independently vary the average value (DC component) and the peak-to-peak amplitude (AC component) of the signal. This makes it possible to test both the absolute sensitivity of the wearable sensor (observing the minimum intensity that the device can detect) and the sensitivity concerning the minimum variation in brightness that it can detect (the minimum value of the peak-to-peak amplitude).

### 2.4. Signal Transmission Scheme

**Figure 4:**
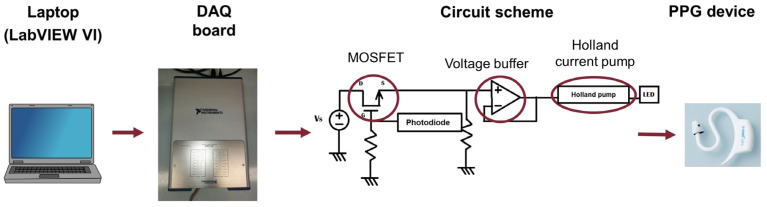
Scheme of the transmission of the PPG signal.

The signal transmission scheme is shown in Figure 4. First, the signal is synthesized using the LabVIEW software, starting from a file of values relating to a photoplethysmographic signal; subsequently, it is generated using a DAQ board and transmitted to the circuit. The final transmission of the signal occurs via an LED on the board, whose light intensity will vary proportionally to the variations in the incoming PPG signal and will be detected by the photodetector present on the PPG device.

In particular, a Howland current pump [26] was used in the circuit to convert the input voltage signal into the current signal that will drive the LED. Furthermore, the board sends the signal only in response to the switching on of the device LEDs; therefore, it is also necessary to use a photodiode that detects changes in brightness connected to a MOSFET, which, acting like a switch, enables signal transmission based on the detected light intensity. A voltage buffer between the MOSFET and the Howland current pump is used to decouple the two systems.

## 3. Electronic Board Design and Implementation

### 3.1. Electronic Board Design

**Figure 5:**
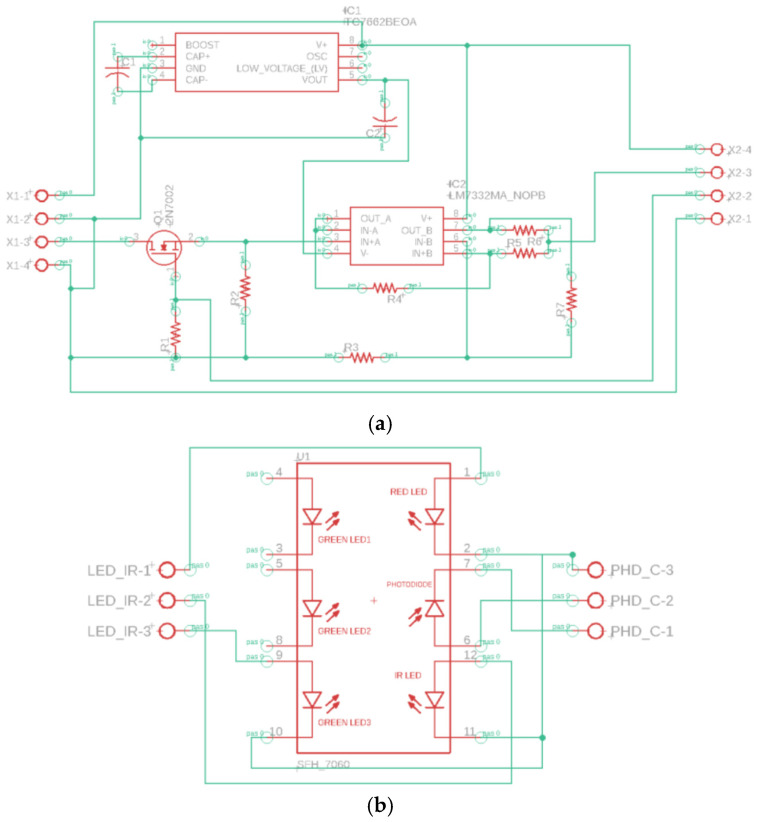
Eagle circuit scheme of PCB1 (**a**) and PCB2 (**b**).

The circuit has been divided into two parts: one of the two circuits contains only the integrated circuit with the LEDs and the photodiode (Figure 5b—scheme of PCB2), while the other contains all the other components (Figure 5a—scheme of PCB1); this subdivision was made to reduce the size of the card containing the LEDs as much as possible so that it could be easily placed close to the PPG device to be tested. Both cards were designed using Easily Applicable Graphical Layout Editor (EAGLE 9.2.2) software [27].

Figure 5a shows a four-pin connector (on the left) that will be connected to the DAQ board: the generated signal is connected to terminal 3, the analog output ground is connected to terminal 4, the +5 V voltage is connected to terminal 1, and the reference digital ground is connected to terminal 2. The latter two are used to power the charge pump voltage converter (TC7662B) that generates negative supply voltages starting from positive input values; for this purpose, two capacitors of 10 μF each were inserted. In this case, the voltage converter was used to have two power supply rails (one positive and one negative +/− 5V) for the operational amplifiers (OPAs) contained in the LM7332 component.

On the right side of the schematic, the Howland current pump made up of five resistors (R3, R4, R5, R6, and R7) and one of the two OPAs contained in the LM7332 component are present. The resistor values were chosen based on the current value that must flow on the LED since it is established by the amplitude of the input voltage signal and the 1/R6 transconductance; assuming that the input signal has an amplitude of 4 V and knowing the maximum current value flowing on the LED (equal to 60 mA from the datasheet), a value of R6 equal to 62 Ω was chosen. Since the resistors must respect a certain relationship for the Howland pump to deliver a current independently from the load, the values of the other resistances were consequently all chosen equal to 10 kΩ. The output of the Howland pump is connected to terminal 3 of the output connector. Furthermore, additional components have been inserted. Since the LED needs to emit light only when the LED of the PPG device is on, a photodiode to measure the light intensity and a MOSFET used as a switch are included in the circuit. The photodiode (contained in the SFH7060 integrated circuit in PCB2) is placed in series with a resistor; it is connected to the gate of the MOSFET so that, when a current flows through it (i.e., when the photodiode is illuminated), the voltage on the resistor increases and, consequently, the voltage on the MOSFET gate increases, so that the MOSFET switches to the active state, and the current can reach the LED. In particular, the MOSFET 2N7002 with a gate threshold voltage of 2.1 V is used; starting from this value and knowing the average value of current flowing on the photodiode (1 μA), it is possible to choose the value for the resistance in series with the photodiode itself (R1), which theoretically should be 2.1 MΩ; in practice, the closest available value is 2.7 MΩ. A unity buffer (using the other OPA of the LM7332) was inserted between the MOSFET and the Howland pump to decouple the two systems. As regards the connections of these components, the drain of the MOSFET is connected to terminal 3 of the input connector, which represents the output signal of the DAQ board; the MOSFET gate is connected to terminal 2 of the output connector; terminal 4 of the input connector represents the analog output ground; this was then connected to the digital ground (terminal 2 of the input connector) to have a single reference in the PCB. Finally, in the output connector, the +5 V voltage and the digital ground are connected to terminals 4 and 1, respectively.

In the PCB2 (Figure 5b), the input connector and the SFH7060 integrated circuit are present. The latter is made up of five LEDs and a photodiode; three LEDs emit light in the green band (530 nm), one emits light in the red band (660 nm), and one emits light in the infrared band (950 nm). To have the possibility of carrying out measurements with LEDs that emit in different bands, the output of the Howland pump will be connected to one of the three LEDs. Finally, the photodiode is powered by the +5 V voltage (terminal 1 of the connector on the right) on one side, and on the other, it is connected to terminal 2 of the same connector; this terminal will be connected to the gate of the MOSFET in the PCB1, thus placing the diode in series with the 2.7 MΩ resistor.

### 3.2. Electronic Board Implementation

**Figure 6:**
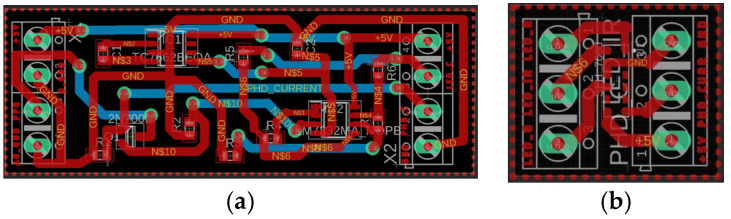
Layout of PCB1 (**a**) and PCB2 (**b**).

After creating the two schematics using the Eagle software, the two layouts were implemented using a double-layer configuration (Figure 6). The red tracks are part of the top layer, while the blue tracks belong to the bottom layer. The overall dimensions of the boards are approximately 6.7 cm × 2.4 cm for PCB1 and 2 cm × 1.7 cm for PCB2.

**Figure 7:**
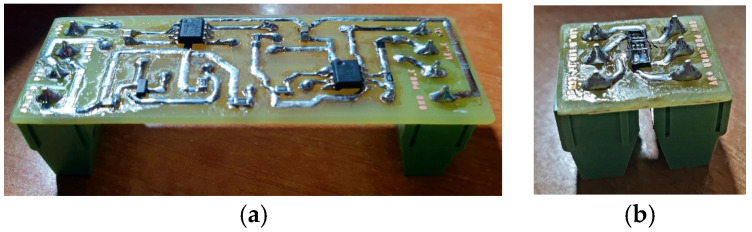
Picture of PCB1 (**a**) and PCB2 (**b**).

Figure 7 shows pictures of the two manufactured boards after the component soldering.

**Figure 8:**
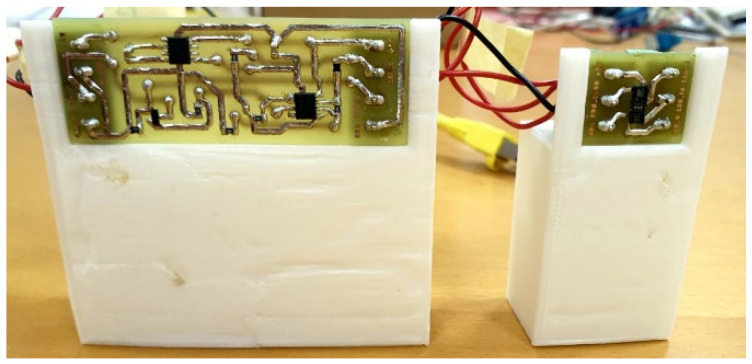
PCB1 and PCB2 mounted on their supports made by 3D printing.

To ensure the reproducibility of the measurements, the circuit boards and the PPG device were placed on stable supports. In particular, tailor-made structures (Figure 8) were created using 3D modeling software (Autodesk Fusion 360 [28]) and a 3D printer (Creality CR-10 Smart 2021 [29]) for the two circuit boards.

### 3.3. Electronic Board Operation Test

The electronic boards were tested with a test signal; in particular, four aspects were considered:

1. The value of the transconductance of the Howland current pump;
2. The correct operation in dark conditions;
3. The independence of the current supplied by the Howland pump from the load;
4. The verification of the signal transmission by the LED towards a PPG device.

First of all, the value of the transconductance of the Howland pump, which theoretically should be equal to 0.016 S, should be verified. To this end, the electronic board was connected on one side to the DAQ board (to provide power and the input signal), and on the other side, it was connected to a digital multimeter whose terminals were connected between the output terminal of the current pump and the ground to measure the current that the pump was delivering to the output. In this condition, a DC signal was initially input to the circuit, setting an average value of 1 V and zero peak-to-peak amplitude in the LabVIEW VI, and it was verified that in these conditions, the current value measured by the multimeter was zero (MOSFET as open switch). On the contrary, if the card terminal connected to the MOSFET gate is placed in contact with the 5 V supply voltage, the multimeter measures a current value approximately equal to the expected 16 mA; therefore, the transconductance value of the Howland pump is exactly as designed.

Second, it was verified that the activation of the MOSFET occurs only when expected, i.e., when the photodiode is illuminated by the light of the LEDs of the device. Initially, it was observed that when the photodiode was hit by ambient light, the multimeter provided the correct output current value, but in dark conditions, the current intensity still remained significantly different from zero, even if quite far from the one obtained in the “on” state. The modest current intensity that flows through the LED in this condition is probably due to the presence of the high-value resistance to the ground (previously indicated as R2, with a value of 10 kΩ), which precedes the Howland pump; this resistance, being much greater than that at the input of the Howland pump and that at the output of the DAQ board, should send the input voltage to zero when the MOSFET is off. However, its value is too high for the polarization currents of the operational amplifier to generate a non-negligible voltage drop on it, which goes to the input of the Howland pump, and consequently, this delivers a modest current intensity. Therefore, it was decided to make a second circuit identical to the first, except for the value of the resistance to ground (R2), which was lowered to 1 kΩ. In this way, an actual improvement was noticed compared to the previous circuit: with ambient light reduced to a minimum, the LED remains off and only lights up when it is illuminated.

Subsequently, since the Howland pump will have to supply current to different LEDs on the board during the measurements, it was ensured that the current supplied was actually independent of the load, i.e., that it was the same regardless of which LED is connected, considering that LEDs at different wavelengths are characterized by different threshold voltages. To this end, the MOSFET gate was connected to the 5 V supply (so that the switch was always closed), and the output of the Howland pump was connected to a different LED for each test; a digital multimeter was connected in series between the output of the current pump and the LED, and by providing a DC signal with an average value of 1 V as an input, as carried out previously, the following intensity current values were obtained:

- 16.1 mA for the red LED;
- 16.1 mA for the infrared LED;
- 16.0 mA for the green LED.

By setting the same input voltage value, the current supplied to the different LEDs is practically the same; therefore, it can be concluded that the current supplied by the Howland pump is actually independent of the load.

**Figure 9:**
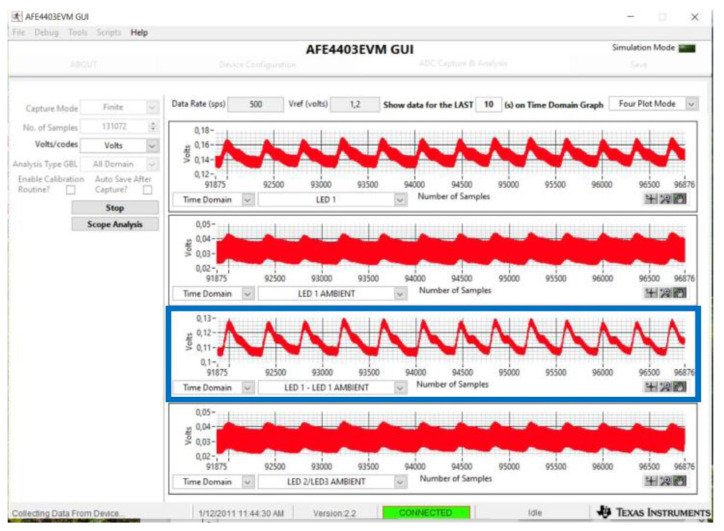
ADC Capture & Analysis section of the AFE4403EVM sensor GUI. The blue box indicates the channel in which the ambient light is subtracted from the signal (LED1-LED1 AMBIENT).

Finally, in order to verify the correct transmission of the signal by the LED, an average value of 0.1 V and a peak-to-peak amplitude of 0.02 V were set in the LabVIEW VI; the signal thus generated was measured using the AFE4403EVM sensor by Texas Instruments [30], which bases its operation on the reflection photoplethysmographic technique. This sensor can be controlled via a graphical interface (GUI). Through the “ADC Capture & Analysis” section (see Figure 9), it is possible to observe the trend of the recorded signals and to evaluate the signal transmitted by the board LED, which can be observed in particular in the blue box (i.e., in the channel in which the ambient light is subtracted from the signal) of Figure 9. In fact, this figure shows the trend of the PPG signal that the card is reproducing; therefore, it can be said that the circuit works correctly as the variations in the input PPG signal are accurately transduced into variations in the luminous intensity of the LED, which lights up considerably only when PCB2 is illuminated by the AFE4403EVM LED.

This is the first significant result since one of the main aims of the work is to implement a circuit that reproduces the PPG signal reflected by the body, starting from values taken from a database.

## 4. Results

This section describes measurements carried out with the Cosinuss° Two device presented in Section 2.2. Measurements carried out on the board are also shown in order to better characterize it and to examine some aspects of the recordings made with the cosinuss° device in more depth.

### 4.1. Measurements with the Cosinuss° Device

#### 4.1.1. Measurement Setup

**Figure 10:**
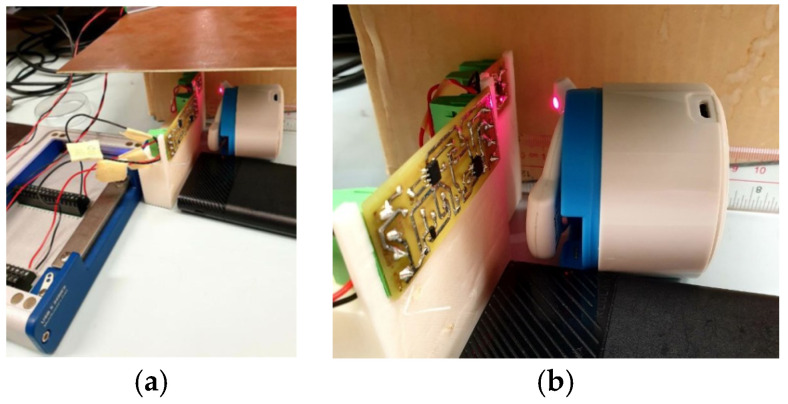
Experimental setup for measurements (**a**) and mutual position between the circuit board and PPG device (**b**).

In order to characterize the device, the circuit elements were arranged as in Figure 10a, with the DAQ board (left), the circuit boards mounted on the respective supports, and the cosinuss° device positioned in front of the board containing the LEDs (PCB2) to maximize the quality of the signal. To minimize ambient light, a metal plate placed above the devices was used. The distance between PCB2 and the device was kept at 2 cm.

The measurement procedure consists of the following steps:

(1) The device is positioned in the ear canal for recording to begin (as the device is temperature-sensitive, this step allows to start the device, which would otherwise remain turned off);
(2) The earphone is kept in this position for about 50 s in order to stabilize the signal;
(3) Then, the device is properly placed in its case, with the optical sensors of both elements at the same height;
(4) After a short transient period (10–20 s), the device begins the correct recording of the PPG signal coming from the board.

Generally, the device is left to record in the final position for about one minute; therefore, considering the initial period in which it is in the ear and the transition periods, the recordings have an average duration of 120–150 s.

The mutual position between the circuit and the device used to carry out all the measurements is represented in Figure 10b.

**Figure 11:**
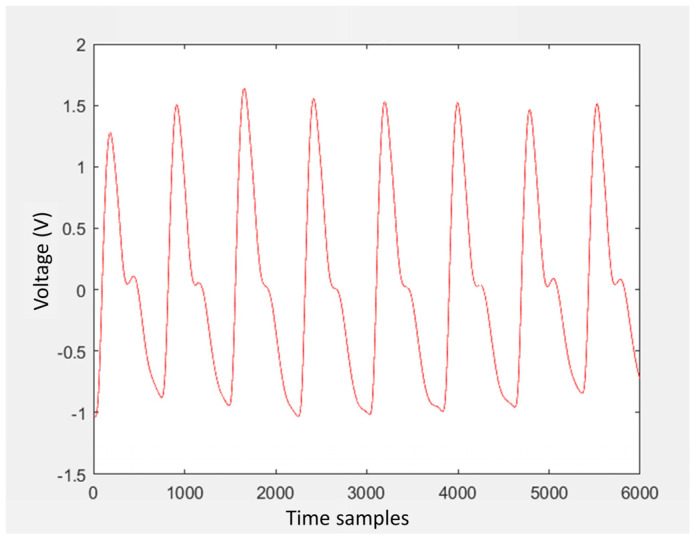
MATLAB plot of the initial PPG signal used to make measurements with the PPG device.

After having verified that the board accurately reproduced the PPG signal through the operation test described in Section 3.3, it was verified that the cosinuss° device was also able to correctly record the signal transmitted by the card. Therefore, the measurements carried out have the aim of both evaluating the correct reception and recording of the signal by the PPG device and testing the PPG device’s performance relating to its sensitivity. The signal used for this purpose is a physiological PPG signal of a healthy subject (Figure 11); it has a duration of approximately 120 s (since it had 120,000 samples acquired with a sampling frequency of 1 kHz). To allow signal generation for more than 2 min, the PPG signal was appropriately replicated in MATLAB so that it had a longer time duration (10 min in total).

Once the signal to be used was selected, it was supplied as an input to the LabVIEW VI; the processed signal will be sent to an output terminal of the DAQ board (selectable by the user), which will, in turn, be connected to the circuit, thus modulating the light intensity of the LED on the board. In this phase, it is necessary to set the average value and the peak-to-peak amplitude (in voltage) of the signal itself on the LabVIEW VI’s front panel. In order to characterize the device in terms of sensitivity, several measurements were carried out in which both the average value and the peak-to-peak amplitude were varied separately. The evaluations of the effects of these variations will be based on the analysis of the graphs of the PPG signal recorded by the device, which can be viewed on the cosinuss° Health platform. In this web interface, it is actually possible to check both the PPG signal recorded in the red and infrared wavelengths and the heart rate and signal quality index, which were taken as a reference to choose the signal section to display; in particular, approximately 20 s of the recording with a better-quality index (between 60 and 80) were considered.

In the following section, results relating to the variation of the signal average value and the peak-to-peak amplitude are illustrated.

#### 4.1.2. Signal Average Value Variation

The signal average value was varied for the following purposes:

1. To find the maximum average value of the signal that the earphone can record correctly and above which the device begins to saturate;
2. To find the minimum average value of the signal that the earphone is able to record correctly and below which the recorded signal can no longer be ascribable to a PPG signal.

**Figure 12:**
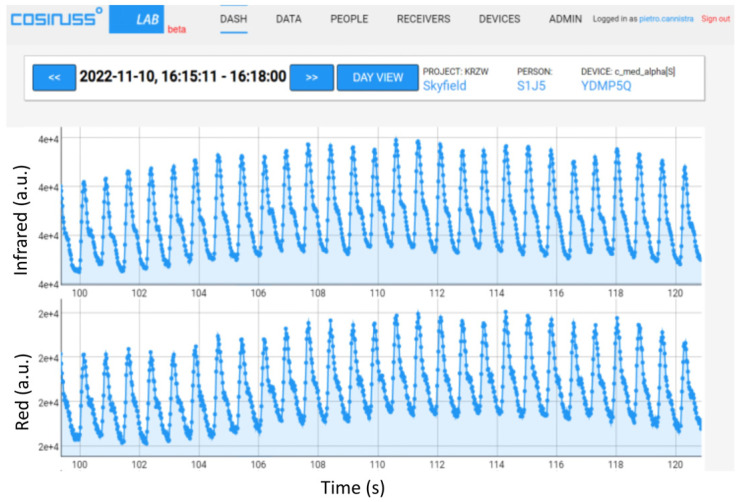
PPG signals recorded by the device, providing an input PPG signal with an average value of 0.1 V and peak-to-peak amplitude of 0.02 V with the red LED on.

These two points have been analyzed for both the red and infrared LEDs since the cosinuss device uses both LEDs and records PPG signals of both wavelengths. Initially, a PPG signal with an average value of 0.1 V with a peak-to-peak amplitude of 0.02 V was used, verifying that the cosinuss device received and recorded it correctly. The cosinuss° LAB Interface is shown in Figure 12 with the two recorded signals.

It is important to note here that in the figure caption “red LED on” refers to the optical simulator LED. In fact, the developed optical simulator is based on the SFH7060 Osram Opto BioMon Sensor and comprises infrared, red, and green emitters. Here, it was considered of interest to use both the red LED and the infrared LED present on the board, which were used alternatively to evaluate the sensitivity of the cosinuss° device to the two wavelengths. However, the cosinuss device always powers both LEDs and expects to receive the two different radiations accordingly. Since the receiving photodiode is wideband, it cannot distinguish between red and infrared and, therefore, records the received red light as infrared (and vice versa) simply on the basis of the wavelength it is expected to receive. Basically, the two recordings labeled as red or infrared are actually the same (the slightly different levels are due to the different gain the cosinuss device uses for receiving the two expected wavelengths).

**Figure 13:**
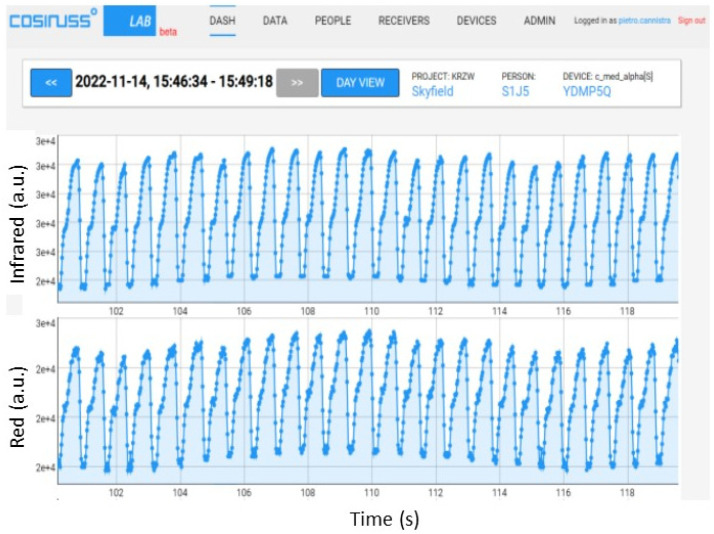
PPG signals recorded by the device, providing an input PPG signal with an average value of 0.8 V and peak-to-peak amplitude equal to 0.2 V with the red LED on.

**Figure 14:**
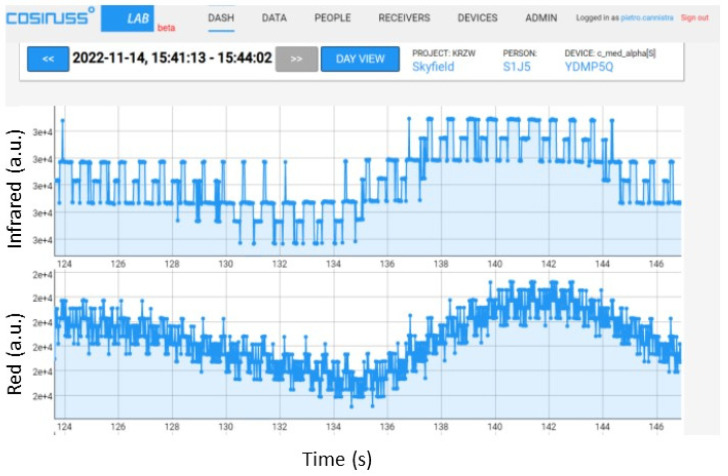
PPG signals recorded by the device, providing an input PPG signal with an average value of 0.85 V and peak-to-peak amplitude equal to 0.2 V with the red LED on.

To identify the limit value beyond which the device saturates, the average value was properly increased (maintaining the peak value equal to 1/5 of the average value). The resulting PPG signal trends recorded by the device are shown below, specifying the average value and the peak-to-peak amplitude of the signal input to the circuit; in particular, some significant results are reported in Figure 13 and Figure 14. Figure 13 shows that, with the same peak-to-peak value (equal to 0.2 V), by sending a PPG signal with an average value of 0.8 V to the device, it manages to record it quite well; instead, a slightly higher average value (0.85 V) causes saturation of the device. In fact, in the recorded signal, there is no longer any trace of the photoplethysmographic waveform (see Figure 14).

**Figure 15:**
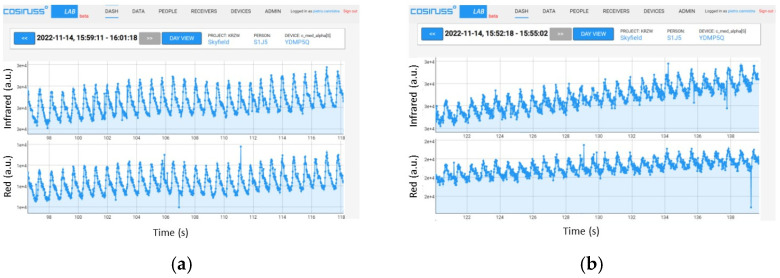
PPG signals recorded by the device, providing an input PPG signal with an average value of 0.03 V and a peak-to-peak amplitude of 0.006 V (**a**) and a signal with an average value of 0.01 V and a peak-to-peak amplitude of equal at 0.002 V (**b**) with the red LED on.

**Figure 16:**
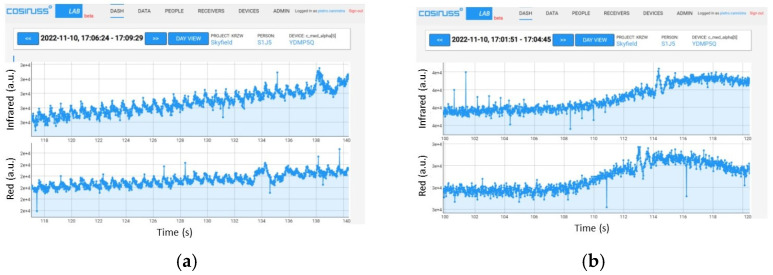
PPG signals recorded by the device, providing an input PPG signal with an average value of 0.008 V and a peak-to-peak amplitude of 0.002 V (**a**) and a signal with an average value of 0.005 V and a peak-to-peak amplitude of the same at 0.001 V (**b**) with the red LED on.

Subsequently, the average value of the signal was gradually decreased (and consequently, the peak-to-peak value, as already explained) to find the device sensitivity threshold. Figure 15 shows some significant trends of the PPG signal recorded by the sensor, with an average value and the peak-to-peak amplitude of the signal input to the circuit specified in the figure captions. From the figures, it can be seen that, as expected, by significantly decreasing the average value of the input PPG signal (which corresponds to a lower light intensity of the LED), the recorded signal appears increasingly noisy. If the input signal has an average value of 0.008 V (Figure 16a), the shape of the PPG can still be seen, while dropping to a value of 0.005 V (Figure 16b), the signal is completely overwhelmed by the noise.

The measurements were repeated by turning on the infrared LED on the board. For the sake of brevity, summary tables summarize the results of the variation of the average value of the input signal, respectively, with the red (Table 1) and with the infrared (Table 2) LEDs turned on.

#### 4.1.3. Results of the Variation of the Signal Peak-to-Peak Amplitude

In this section, the peak-to-peak amplitude signal has been varied. In particular, the response obtained with a PPG signal with an average value of 0.1 V and a peak-to-peak amplitude of 0.02 V, both with the red LED and the infrared LED, was considered. The peak-to-peak amplitude was varied in only one direction, i.e., it was decreased until the minimum value for which the device can record correctly and below which the recorded signal is not more ascribable to a PPG signal was reached.

**Figure 17:**
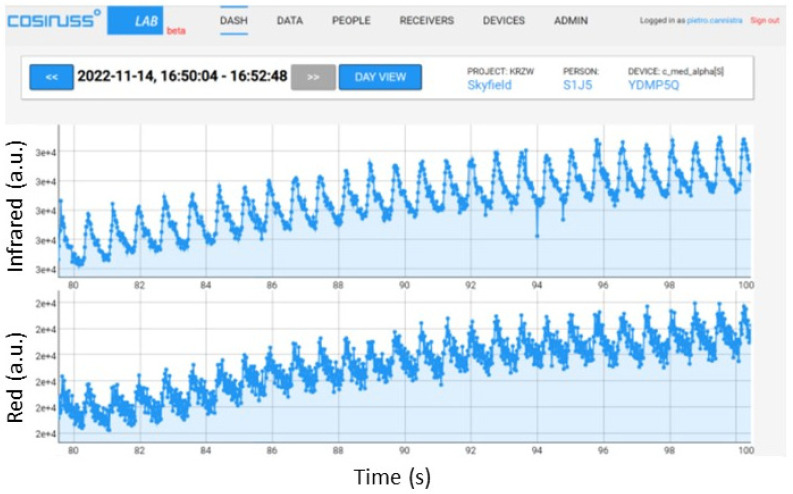
PPG signals recorded by the device, providing an input PPG signal with an average value of 0.1 V and peak-to-peak amplitude of 0.0075 V with the red LED on.

**Figure 18:**
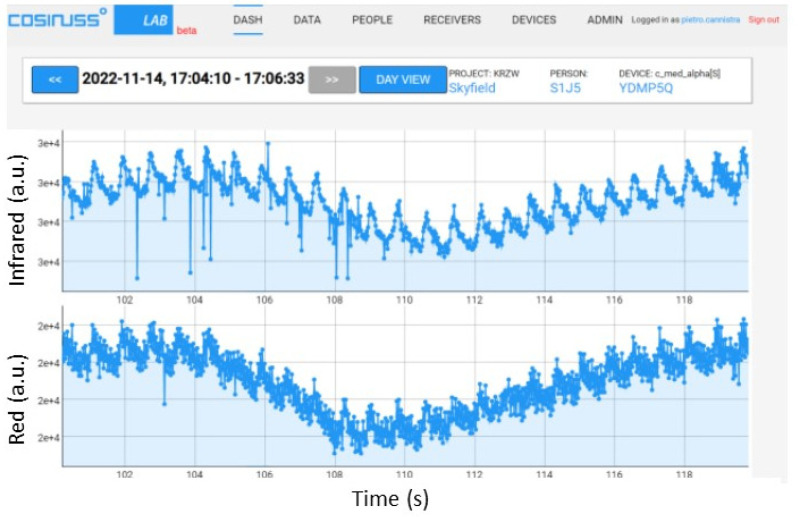
PPG signals recorded by the device, providing an input PPG signal with an average value of 0.1 V and peak-to-peak amplitude of 0.005 V with red LED on.

**Figure 19:**
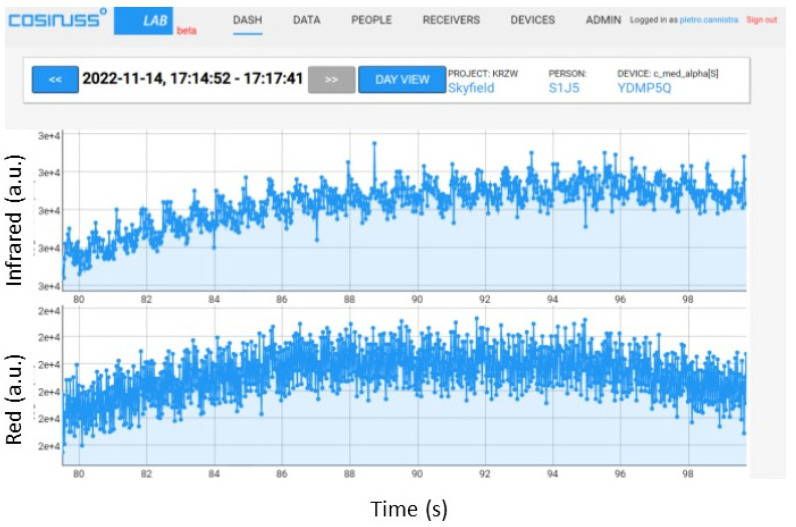
PPG signals recorded by the device, providing an input PPG signal with an average value of 0.1 V and peak-to-peak amplitude of 0.001 V with a red LED on.

Both the red and infrared LEDs present on the board were used for these tests. To begin, the most significant trends obtained by turning on the red LED of the board, maintaining the average value of the input signal at 0.1 V, and gradually decreasing the peak-to-peak amplitude from 0.0075 V to 0.001 V are shown (Figure 17, Figure 18 and Figure 19).

It is possible to notice that with a peak-to-peak amplitude equal to 1/20 of the average value, the signal can still be distinguished quite well (Figure 18), but by further lowering this value to 1/100 of the average value, the signal begins to be no longer recognizable (Figure 19).

The same experimental tests have been conducted with the infrared LED. Below are summary tables showing the results of varying the peak-to-peak amplitude of the input signal with the red (Table 3) and infrared (Table 4) LEDs turned on, respectively.

For the case of the infrared LED, it is noted that a peak-to-peak amplitude value of the signal equal to 1/20 of its average value leads to a recorded signal that can no longer be ascribable to a PPG signal.

#### 4.1.4. Results of Signal Frequency Variation

**Figure 20:**
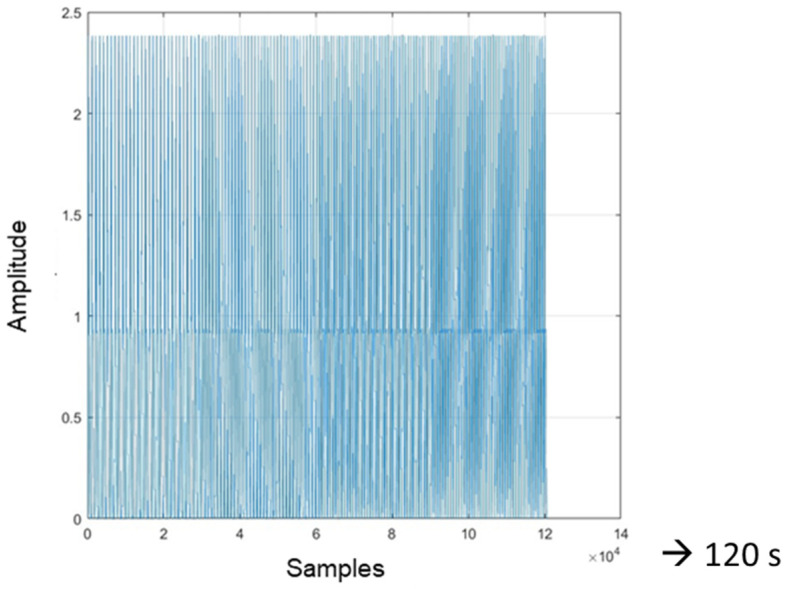
Sequence of 4 PPG signals.

**Figure 21:**
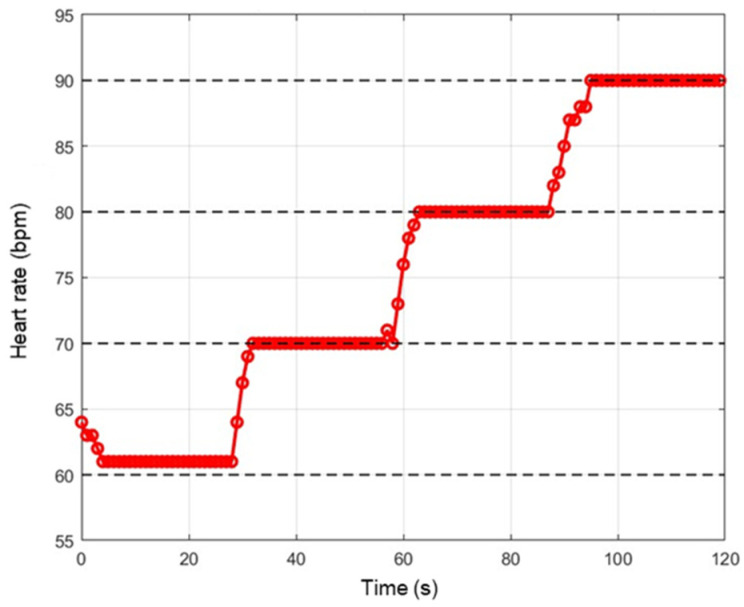
Trend of the heart rate detected by the device by supplying a variable frequency PPG signal as input.

To evaluate the sensitivity of the device to the signal frequency (i.e., heart rate) variation, a PPG signal was specially created with MATLAB consisting of a series of four PPG signals, each with its own fundamental frequency, respectively, of 1 Hz (60 bpm), 1.167 Hz (70 bpm), 1.333 Hz (80 bpm), and 1.5 Hz (90 bpm), with each lasting 30 s for a total duration of 120 s (Figure 20). As can be noted in Figure 21, the device takes about 3 s to switch from one average value to another; therefore, the transition time is satisfactory.

#### 4.1.5. Evaluation of LED Light Power

All results reported in the previous sub-sections refer to the input voltage level at PCB1 (i.e., the input voltage level at the current pump). Since the transconductance value of the pump is known, these voltage values directly translate to current values feeding PCB2 LEDs. However, the most important thing is to relate such voltage values to the actual optical power emitted by the LEDs and impinging on the PPG device being tested. To this end, in order to quantify the LED emitted light intensity, with a certain average voltage value set at the input, measurements were carried by an optical power meter, in particular, the “Coherent Fieldmaster GS Laser Power and Energy Analyzer” [24] (photosensitive area 19 mm^2^). To measure the transmitted optical power, the MOSFET gate is connected to 5 V. The measurement procedure used is the following:

- Setting the wavelength of interest on the power meter;
- Evaluation of the power recorded in the presence of ambient light;
- Positioning the LED and the power meter sensor in front of each other;
- Sending the PPG signal to the circuit with consequent switching on of the LED.

Measurements were carried out by sequentially turning on the two red and infrared LEDs on the board; in both cases, first, the power value caused by the ambient light was measured and found to be negligible, being much lower than the other recorded values. The obtained results are summarized in Table 5.

As a first step, the measured power values were compared with those reported on the datasheet of the SFH7060 component. In particular, on the SFH7060 datasheet, a light power equal to 6.4 mW for the red LED and 5.3 mW for the infrared LED is specified, given a certain current intensity (20 mA). In Table 5, it is shown that a value of 16.1 mA results in around 1 mW for the red LED and considerably less for the infrared LED. This has been attributed to the limited sensitive area of the power meter sensor, which is unable to capture all the emitted light; moreover, given that the sensors are placed a certain distance from the board, only a small part of the LED power is able due to a large beam width of the emitted radiation.

In the first lines of Table 5, the power values sent by the LEDs relating to the sensitivity threshold of the cosinuss° device can be observed (PPG signal overwhelmed by noise, see Figure 16). In particular, in these tests, it was considered that the measurements performed on the cosinuss° earphone with the two LEDs had different results, so the lowest average voltage value was set only when using the red LED.

Since the power emitted was underestimated compared to the values reported in the datasheets, it was decided to carry out these measurements using another optical power meter, the Newport model 1830-C [25]. In particular, the sensor of this new power meter is characterized by a larger sensitive area than the previous one (100 mm^2^). This effectively translates into a greater quantity of radiation that the sensor can capture, providing a doubled reading compared to the previous sensor. However, values are still quite far from those reported in the datasheet; therefore, it can be concluded that a part of the radiation is not reaching the sensor, and the power is still underestimated. Considering this, it has been estimated that the minimum voltage thresholds for red and infrared are a few tens of μW and tens of μW, respectively. As regards the voltage limits beyond which the device goes into saturation, the corresponding measured emitted light power values are in the order of a few mW for red and mW for infrared.

## 5. Discussion

In this paper, a promising method for preventing and reducing the risk of cardiovascular diseases, i.e., the photoplethysmographic technique, has been explored. In particular, a PPG ear sensor was characterized, the cosinuss° c-med° alpha, which measures heart rate, oxygen saturation, and body temperature starting from a PPG signal. A circuit has been designed and implemented that reproduces the PPG signal reflected by the body, starting from values taken from a database. This approach has some undeniable advantages compared to the system previously proposed in [18], as it allows the testing of different PPG devices, both to make a comparison between them and/or to test the same device with signals with different characteristics. This opens up the possibility of diagnosing and/or predicting pathologies by comparing different medical sensors. Moreover, the system presented in [18] presents some critical issues; for example, in the comparison of different devices, they will be positioned at slightly different points; furthermore, the system that produces the pressure wave generates a periodic signal that is not exactly comparable to a physiological signal, so it is not possible to carry out any clinical diagnosis on it. Through the optical simulator, it is possible to test a device with a huge number of signals coming from subjects present in medical databases. However, once the PPG device is characterized, its functioning will be verified on human subjects. In this case, motion artifacts could be present, but we expect that the estimated performance will still be confirmed.

The PPG signal taken from a database is processed via software and sent to the circuit via a DAQ card. In this way, the light intensity of the LEDs will be directly proportional to the voltage value set at the input and will vary in accordance with the variations in the input PPG signal. Furthermore, the optical simulator must send the signal only in response to the switching on of the device under test LEDs, which is why it was also necessary to use a photodiode that detected changes in brightness connected to a switch (a MOSFET), thus determining whether to send the signal or not.

The correct replica of the input signal by the electronic board and the opening/closing mechanism of the switch were verified both using a Texas Instruments sensor and using the cosinuss° earphone. The latter was then used to validate the functioning of the designed electronic board. Moreover, the computation of the heart rate was examined to evaluate its sensitivity concerning the variation in frequency; by providing an input PPG signal that varies its frequency instantly, it was found that the device takes approximately 3 s to move from one average heart rate value to the next one.

Moreover, the absolute sensitivity of the cosinuss° earphone was also tested, varying the average value and peak-to-peak amplitude of the signal as desired. The PPG signal of a healthy subject was then transmitted to the device by turning on the red and infrared LEDs on the board separately, finding slightly different results in the two cases: for the first LED, the range in which the c-med° alpha earphone records a signal of good quality is slightly more extended, going from an average voltage value of 0.03 V to 0.8 V, while for the infrared LED, this range extends from 0.07 V to 0.7 V. On the contrary, by varying only the peak-to-peak amplitude of the signal, no notable differences were found between the two LEDs. It is worth noting that, while the cosinuss° earphone turns on both its LEDs to send the signal, the radiation it receives back is all in a certain wavelength range (red or infrared); this is probably a limitation of the designed optical simulator, and as a future development, the simultaneous switching on of the two LEDs had to be considered. Additionally, a further improvement could be the integration of a microcontroller into the board so that we no longer have to depend on the DAQ board to generate the input signal, a quite bulky instrument only available in a laboratory.

Moreover, it was considered of interest to characterize the threshold values in terms of light intensity emitted by the LED and consequently received by the device. Therefore, in order to evaluate the correspondence between the voltage level set at the input and the order of magnitude of the light intensity that the LED on the board emits, optical power measurements were carried out. For these measurements, the power values provided are underestimated; therefore, it can be stated that the corresponding minimum voltage thresholds for red and infrared are of the order of a few tens of μW and tens of μW, respectively, while emitted light power values beyond which the device goes into saturation are of the order of a few mW for red and mW for infrared.

## 6. Conclusions

The characterization of the cosinuss device previously presented shows the potential of the proposed optical PPG emulator as a tool for testing the functionality of new PPG designs and comparing the performances of different PPG sensors.

The most interesting feature, however, is the possibility to inject a PPG signal taken from a clinical database containing data from subjects affected by specific cardiac diseases, thus testing the diagnostic capabilities of the devices before performing actual clinical trials, which are time-consuming and require specific certifications and protocol approval. This could result in an enhanced time-to-market, which would allow a more straightforward development of new wearable technologies that represent a key enabling technology for e-health deployment.

## Figures and Tables

**Table 1:** Results for the variation of the average value with the red LED on.

Average Value (V)	Peak-to-Peak Amplitude (V)	Distance (cm)	Recorded Signal
0.005	0.001	2	signal overwhelmed by noise
0.008	0.002	2	very weak but recognizable PPG signal
0.03	0.006	2	robust PPG signal
0.8	0.2	2	still recognizable signal
0.85	0.2	2	device saturation

**Table 2:** Results for the variation of the average value with the infrared LED turned on.

Average Value (V)	Peak-to-Peak Amplitude (V)	Distance (cm)	Recorded Signal
0.008	0.002	2	signal overwhelmed by noise
0.01	0.002	2	very weak but recognizable PPG signal
0.07	0.01	2	robust PPG signal
0.7	0.2	2	still recognizable signal
0.75	0.2	2	device saturation

**Table 3:** Results for peak-to-peak amplitude variation with the red LED on.

Average Value (V)	Peak-to-Peak Amplitude (V)	Distance (cm)	Recorded Signal
**0.1**	0.001	2	signal overwhelmed by noise
**0.1**	0.005	2	very weak but recognizable PPG signal
**0.1**	0.0075	2	robust PPG signal

**Table 4:** Results for peak-to-peak amplitude variation with the infrared LED on.

Average Value (V)	Peak-to-Peak Amplitude (V)	Distance (cm)	Recorded Signal
**0.1**	0.005	2	signal overwhelmed by noise
**0.1**	0.0075	2	robust PPG signal

**Table 5:** Results for peak-to-peak amplitude variation for the infrared LED.

Average Value (V)	LED Current (mA)	Measured Power of Red LED (µW)	Measured Power of Infrared LED (µW)
**0.008**	0.13	1.77	//
**0.01**	0.16	2.66	0.513
**0.03**	0.48	15.2	2.51
**0.07**	1.1	47.8	8.30
**0.1**	1.6	74.5	13.3
**0.2**	3.2	168	31.8
**0.3**	4.8	264	51.6
**0.4**	6.5	362	72.2
**0.5**	8.1	460	93.1
**0.6**	9.7	558	114
**0.7**	11.3	656	136
**0.8**	12.9	755	157
**0.9**	14.5	853	179
**1**	16.1	954	200

## Data Availability

Data are contained within the article.
